# Older Adults’ Experiences of Telephone-Delivered Behavioral Activation with Mental Imagery as a Treatment for Depression During the COVID-19 Pandemic: A Qualitative Study

**DOI:** 10.3390/bs15060807

**Published:** 2025-06-12

**Authors:** Johnny Pellas, Mattias Damberg, Fritz Renner, Julie L. Ji, Marie Kivi

**Affiliations:** 1 Department of Public Health and Caring Sciences, Uppsala University, 751 22 Uppsala, Sweden; mattias.damberg@uu.se; 2 Centre for Clinical Research, Uppsala University, Västmanland County Hospital, 721 89 Västerås, Sweden; 3 Department of Clinical Psychology and Psychotherapy, University of Freiburg, D-79106 Freiburg, Germany; fritz.renner@psychologie.uni-freiburg.de; 4 School of Psychology, University of Plymouth, Plymouth PL4 8AA, UK; julie.ji@plymouth.ac.uk; 5 Department of Psychology, University of Gothenburg, 405 30 Gothenburg, Sweden; marie.kivi@goteborgsregionen.se

**Keywords:** aging, elderly, CBT, geriatric, late life, qualitative

## Abstract

The COVID-19 pandemic prompted the use of telehealth interventions for treating depression in older adults. We conducted a pilot study of a telephone-based brief psychological intervention, Behavioral Activation with Mental Imagery (BA-MI), for the treatment of depression in isolated older adults during the COVID-19 pandemic. We achieved promising results regarding a reduction in depressive symptoms. The purpose of the present study was to investigate the participants’ experiences of the intervention and provide insight into how the intervention could be improved. Fourteen participants aged 67–85 years that completed the BA-MI intervention were purposively selected and interviewed. The interviews were analyzed using thematic analysis with a descriptive phenomenological approach. BA was experienced as a good way of increasing activities and improving mood, but the opinions on MI were divided. Telephone delivery reduced barriers due to pandemic restrictions but felt less personal and lacking non-verbal communication. Being recognized and talking to a therapist every week was described as healing. When using manual-based psychological interventions, one should aim to make them as person-centered as possible by making room for the patients as individuals with both a past and a present, rather than just focusing on intervention delivery.

## 1. Introduction

In older adult populations worldwide, one-third report depressive symptoms (Hu et al., 2022), and one in ten suffer from a depressive disorder (Abdoli et al., 2022). Depression is considered one of the leading causes of the global health burden across the lifespan (Ferrari et al., 2022). In the beginning of 2020, Coronavirus disease 2019 (COVID-19) spread across the globe and was classified as a pandemic. Because of the high mortality rate associated with the disease, especially in older adults, many countries adopted a strategy of social distancing, urging older and vulnerable persons to limit close contact with others. The COVID-19 pandemic increased the rates of depressive symptoms in older adults (Tyler et al., 2021), prompting the need for effective treatments delivered remotely to ensure adherence to the pandemic restrictions. A common example of such treatments is telehealth interventions; that is, interventions that are delivered via the internet, videoconferencing, or the telephone. Telehealth interventions for mental health issues have been used for decades and previous studies on telehealth interventions for depression delivered via the Internet (Xiang et al., 2020), videoconferencing (Egede et al., 2015), and telephone (Castro et al., 2020) show effects comparable to psychotherapy that is delivered face to face. A handful of studies on telehealth interventions for depression in older adults were conducted during the COVID-19 pandemic, showing promising results with significant reductions in depressive symptoms and loneliness (Silva et al., 2023). One of these studies was conducted by our research group, a pilot randomized clinical trial investigating the feasibility, acceptability, and preliminary efficacy of a brief behavioral activation intervention augmented with mental imagery (BA-MI) for older adults with depression, living in isolation due to the COVID-19 pandemic (Pellas et al., 2022). The BA-MI intervention was based on a four-session behavioral activation (BA) treatment (Funderburk et al., 2020; Funderburk et al., 2021) augmented by a mental imagery (MI) exercise during sessions two and three (Pellas et al., 2022). BA is a brief psychological treatment for depression focusing on increasing enjoyable, important and meaningful activities, and decreasing depressive activities (Dimidjian et al., 2011). BA is effective for treating depression in adults in general (Cuijpers et al., 2007), and accumulating evidence shows that BA is also effective in older adults (Orgeta et al., 2017). Shearsmith et al. (2023) conducted a deductive qualitative study of a pilot BA intervention during the COVID-19 pandemic (Gilbody et al., 2021) using the theoretical framework of acceptability. The results showed that older adults described BA as a useful way of making changes and achieving their goals during the pandemic and that their self-efficacy developed over time, although the pandemic restrictions were described as a limitation for activity planning (Shearsmith et al., 2023). The intervention was understood in general, but less so in older adults with low mood. The participants stated that they valued social contact with their therapists. It is important to note that most of the participants in the pilot study had low levels of depressive symptoms at baseline, and most of them were below the cutoff for suspected depression (Gilbody et al., 2021).

MI is defined as a perceptual experience without external sensory input (Pearson et al., 2015), e.g., seeing something in your mind’s eye. In our study, visual MI was applied by choosing scheduled activities from the BA component and guiding the participant through a mental simulation of performing the activity, which involved visualizing themselves engaging in the activity at realistic locations, settings, and times, focusing on the positive aspects of the activity and the potential rewards. Mental imagery (MI) can be described as a “motivational amplifier” when used in conjunction with BA, increasing the likelihood of performing scheduled activities (Heise et al., 2022; Ji et al., 2021; Renner et al., 2019). In our pilot RCT of BA-MI, we adapted and used the MI exercise described by Renner et al. (2019), where participants were asked to vividly imagine future activities. To our knowledge, there are no qualitative studies on MI for older adults, nor have we found any qualitative studies on the combination of MI and BA for the treatment of depression.

As mentioned, the BA-MI intervention consisted of four sessions (Pellas et al., 2022). In session 1, the therapists provided information about depression and BA to the participants. An activity log was also introduced, where the participants were asked to write down their daily activities for one week and rate how they felt after performing the activities. Highly motivated participants were also asked to plan one or more additional activities for the coming week. In session 2, the activity log was reviewed, with the purpose of investigating the connection between activities and mood. After that, the goals and values of the individual patient were discussed, which led to the planning of enjoyable, meaningful, and important activities for the coming week. An activity list was also presented to the participants as a source of inspiration, consisting of activities that were possible to perform in line with the pandemic restrictions. Subsequently, an MI exercise was performed, where the participants were guided through a visualization of performing one of the planned activities. In session 3, the planned activities were reviewed and, if necessary, problem solving was applied. After that, new activities were planned for the coming week and one of the planned activities was used for an MI exercise. Session 4 also started with a review of the previous week’s planned activities, and problem solving was applied if needed. After that, the treatment was reviewed in its entirety, and a plan for continuing BA was made, after which the treatment ended.

The results from our pilot study of BA-MI showed a significant decrease in depressive symptoms in the intervention group compared to the control group, with a large effect size post-treatment (Pellas et al., 2022), and a medium effect size 6 months post-intervention (Pellas et al., 2023). Although most participants were satisfied with the intervention overall (Pellas et al., 2022), we lack in-depth information on participants’ experiences with each component of the intervention, and if and how the treatment could be improved for a full-scale clinical trial. The aim of the present study is to investigate patients’ experiences of the telephone-based BA-MI intervention as a treatment for depression during the COVID-19 pandemic.

## 2. Materials and Methods

### 2.1. Design

Our study was a qualitative study embedded in the pilot RCT, using a descriptive phenomenological approach based on individual interviews. Descriptive phenomenology is aimed at understanding patterns of meaning described in the lived experiences of the participants (Sundler et al., 2019), and this enabled us to evaluate aspects of the intervention that the quantitative pilot study and long-term study could not. The study received ethical approval from the Swedish Ethical Review Authority (ID 2020-02079), and the pilot RCT was preregistered with ClinicalTrials.gov (ID NCT04508868).

### 2.2. Participants

The participants in the pilot RCT of BA-MI were recruited through advertisements in local newspapers and posters in primary health clinics. From the participants in the pilot RCT, a purposive sampling was performed to ensure variability with regard to sex, age, geography (rural/urban), and treatment effect, resulting in fourteen participants that had previously received the BA-MI intervention. Participants were selected by the study coordinator in collaboration with the study’s therapists according to the criteria for the purposive sampling after all participants in the pilot RCT had completed their treatment. The mean age was 73.7 years, ranging between 67 and 85 years. Eleven participants were women (79%) and three were men (21%). Seven participants lived alone; seven lived with a partner. At inclusion, eleven participants met diagnostic criteria for major depressive episode (79%), while three met criteria for a minor depressive episode (21%). The demographic characteristics in this study were comparable to the pilot RCT, where the mean age in the treatment group was 75 years with 80% females and 20% males, 45% lived with a partner and 65% lived alone, and 80% met the diagnostic criteria for a major depressive episode. All participants received oral study information by telephone as well as written study information sent by mail. All participants provided written informed consent that was sent by mail.

### 2.3. Data Collection

Individual, semi-structured interviews were conducted via telephone according to an interview guide (see Table 1 for an English translation). All interviewers were psychologists participating as therapists in the pilot study during their one-year internship before obtaining their license. To minimize bias and to enable the participants to be frank about their experiences, no participant was interviewed by their own therapist or by the study coordinator (conducting assessment at enrolment). All interviews were conducted between December 2020 and January 2021, one to three months post-intervention. The interviews lasted 13 to 54 min, with a mean time of 29 min. The interviews were recorded on a digital voice recorder (Olympus VN-741PC, Olympus, Tokyo, Japan), then transcribed verbatim and checked for accuracy by each interviewer.

### 2.4. Analysis

The interviews were analyzed using thematic analysis (TA) with a descriptive phenomenological approach (Sundler et al., 2019). The analysis was conducted in an inductive manner, i.e., data-driven rather than hypothesis-driven. The interviews were analyzed by two researchers, JP and MK, who are licensed clinical psychologists certified in cognitive behavioral therapy (CBT) with experience in geropsychology, with 13 and 18 years of experience as licensed clinical psychologists, respectively. The researchers started by familiarizing themselves with the data by reading the interview texts several times, exploring the experiences described by the participants. Next, meanings in these experiences were identified and organized into patterns by the researchers using Microsoft Excel, and from these patterns, preliminary themes and sub-themes were developed by each researcher independently. The researchers then met to discuss their findings and preliminary themes, followed by additional meetings to redefine, name, and describe the final themes. In line with the recommendations by Sundler et al. (2019), the researchers strived to be open to the life experiences and phenomena described by the participants to uncover their lived experiences. To achieve this, the researchers tried to keep an open and reflective attitude towards their own pre-understandings by questioning the interpretations made throughout the analysis. Thus, several reiterations of the analysis were made, reflecting the dynamic process of developing themes within a more reflexive tradition of TA (Braun & Clarke, 2006, 2022).

## 3. Results

Five themes were defined based on the analysis: (1) the acceptability of BA as concept in relation to depression; (2) MI as a motivator or irritator; (3) telephone contact being similar to face-to-face contact, but not the same; (4) the importance of being seen as a whole person; and (5) the power of being recognized. See Table 2 for a list of themes and sub-themes, and a brief summary of the findings related to each sub-theme.

### 3.1. Acceptability of BA as a Concept in Relation to Depression

#### 3.1.1. Diary Increased Self-Awareness

In between session one and two, the participants were asked to keep an activity diary for seven days, and register their activities and mood at two-hour intervals. The purpose of the diary was to investigate the connection between behavior and mood, and to serve as a starting point for activity planning.

Generally, participants said that the diary had given them insights into their own behavior and their mood, and thereby increased their self-awareness. Some participants described how the diary gave them concrete information about previous activities:

*“…the diary was very good because I could see in retrospect what I had done and how many contacts I actually had.”*

—Woman in her 70s

Other participants also said that the diary provided them with information about the connection between their previous activities and their mood:

*“I thought the activity diary was very good because I could, like, see what I did all day and how much time it took and how valuable I thought it was or how important it was or things like that. That was how I could see myself, what I had done and why I did it and if there was any value in it. I think it was the best part.”*

—Woman in her 60s

#### 3.1.2. Activity Planning Made Me More Active

The BA intervention aimed to increase adaptive activities and decrease depressive activities (e.g., avoidance, rumination), and thereby affect mood. To achieve this, the patient and therapist worked together during sessions two and three to devise activities that the patient found to be enjoyable, meaningful, and/or important. In between sessions two–three and three–four, the patients then engaged in these activities following an activity plan and rated their mood after completing the activities.

Most of the participants described how the activity planning made them more active, but there were differences in what this increase in activation was attributed to. Some participants expressed that the mere planning influenced whether the activities were performed:

*“Yes, I believe that I’ve become more determined, not just saying that perhaps we will do something, but rather ‘tomorrow we will do it.’”*

—Woman in her 60s

Other participants said that seeing the planned activities in writing made them inspired and motivated:

*“Yes, I see the activities in front of me and what I’m going to do and feel like “on Friday I’m going to do this and that’ll be fun.’”*

—Woman in her 60s

#### 3.1.3. Follow-Up Increased Motivation

During sessions three and four, the therapist followed up on the activity planning by asking if the activities had been completed and, if so, if they had had any impact on the patients’ mood. One participant expressed that following up with specific goals she had achieved increased her motivation to perform the activities:

*“And then you got some assignments, that uh, you had to set a goal to do something. …and then you did it, and you had to tell how you had experienced this goal, and activity, and [setting the goal] that is what made it happen.”*

—Woman in her 70s

Another participant said that sharing the BA plan and following up on the plan with another person, in this case the therapist, increased his commitment and provided an anticipated social reward:

*“I did those things that we agreed upon, but at the same time I really looked forward to the calls. It really gave an acknowledgement that I could say that ‘I have managed to do this activity because we said so.’”*

—Man in his 70s

#### 3.1.4. Pandemic Restrictions as a Barrier for Activities

The intervention was delivered during fall 2020 and the COVID-19 pandemic, when older adults were recommended to engage in social distancing. This limited the possibility of engaging in several activities, such as face-to-face social interactions, or activities that were postponed due to the recommendations. This barrier for activities was mentioned by the participants:

*“I feel like activities, that’s very hard to talk about now during corona-times and when I can’t be active, I mean, I can go out for a walk, but I can’t participate in things that I’ve participated in before… For me, activity means being out and meeting people, doing things with others.”*

—Woman in her 80s

### 3.2. MI as Motivator or Irritator

To increase the participants’ motivation to complete the planned activities, an MI intervention was performed in sessions two and three. This was conceptualized and practiced in an exercise where the participants were asked to visualize looking at a lemon in close detail, cutting the lemon, and then smelling and tasting it. Next, the participants were asked to perform the same exercise using one of their planned activities and focus on the positive and/or rewarding feelings associated with performing and completing the activity. The statements made by the participants reflect a division where some participants regarded MI as a useful tool and motivator, whereas others could not see the point with MI and, in some cases, felt irritated with the MI interventions.

#### 3.2.1. MI as a Useful Tool

Participants described how the MI exercises made them more focused on the rewarding aspects of their goals and that it helped them perform the activities:

*“Most of what you visualize, makes you succeed at what you do. It’s a matter of finding the positive images of your goals ahead.”*

—Man in his 70s

Other participants described how the MI exercises gave them a deeper understanding of the potential positive effects of the activities:

*“Then it was this mental imagery, with the lemon and all that. If you do it you feel better, you understand it better.”*

—Woman in her 60s

One participant found the MI exercise soothing and said that this gave her a way to escape into another world:

*“First and foremost, when I did this, I thought what is this? I was kind of set on that this will lead to nothing, but then I must say it was very soothing. … That is, I turned inwards, and it felt like a whole different world.”*

—Woman in her 60s

#### 3.2.2. No Point Using MI

One participant said that the MI exercise felt silly, and that it felt very far from her as a person:

*“And the first time I was supposed to imagine holding a lemon, and then cutting the lemon and see what it looked like. And that felt a bit silly actually… It didn’t feel relevant that I should imagine going to the hairdresser or giving away old clothes to charity or looking at a lemon. I think I’m a bit too down to earth for that.”*

—Woman in her 80s

Another participant expressed difficulties with visual mental imagery:

*“Eh it didn’t work so well. I guess it’s hard for me to imagine how something looks, I can’t see it in front of me.”*

—Woman in her 60s

### 3.3. Telephone Contact Being Similar to Face-to-Face Contact, but Not the Same

As the trial was designed to comply with pandemic restrictions and to minimize the risk of virus transmission, both the assessment at enrollment and the interventions were performed via the telephone. And indeed, several participants said that the telephone was an important tool to overcome barriers, but they also said that there were several disadvantages using telephone contact only, such as the loss of non-verbal communication and that the contact felt impersonal. Several participants suggested combining the telephone intervention with an initial session face-to-face.

#### 3.3.1. Telephone Reduced Barriers

One participant said that a meeting in real life would be preferable, but at the same time, that it would have been easier to avoid compared to a telephone call:

*“In one way I would rather sit in a room together with a person… But to be honest, that would have made the threshold higher, that I perhaps hadn’t been able to follow through.”*

—Man in his 60s

Another participant expressed that the telephone became an essential tool for communication during the pandemic, especially as more and more remote communication is taking place via the internet, which can become a barrier:

*“The telephone is fantastic! Yes, because many older persons aren’t online and then that’s not an option for them. For them, the telephone is a lifeline.”*

—Woman in her 70s

#### 3.3.2. Disadvantages with Telephone Contact

One participant expressed that contact over the telephone only makes you miss out on non-verbal communication:

*“I prefer meeting face to face because… digital meetings and telephone meetings are only about the words… and then you miss out on a big part of the other language, the quiet language.”*

—Man in his 70s

Several participants said that telephone contact was less personal than face-to-face contact:

*“It’s always more difficult to talk over the telephone instead of talking face to face… It becomes quite anonymous, you don’t know who you are talking to or… well, I mean, you have a name, but you don’t have a face.”*

—Woman in her 80s

#### 3.3.3. Adding Face-to-Face Contact Could Improve Treatment

Several participants expressed that they were satisfied with telephone contact, but that the treatment would benefit from the addition of at least one session face to face, for example:

*“If it would be possible, if you could have the first meeting face to face, and then the other meetings… A personal contact, a personal encounter cannot be replaced by anything… But then, having the rest over the telephone, that would be excellent!”*

—Woman in her 60s

### 3.4. The Importance of Being Seen as a Whole Person

The BA-MI intervention was a time-limited, first-line intervention focused on increasing participants’ participation in enjoyable, important, and meaningful activities. Several participants expressed an unmet need to talk about possible causes of their depression. Some participants also felt a need for increased person-centeredness, which would involve talking more about themselves as individuals, their emotions, and their previous experiences.

#### 3.4.1. The Need to Discuss Causes of Depression with Therapist

Participants described events in the past that they linked to their depression, and one participant also suggested allocating more time for a discussion of potential causes:

*“Perhaps also the causes, why you feel the way you feel… It [the depression] didn’t come out of the blue, of course there are a lot of things behind it.”*

—Woman in her 80s

Others said that although the pandemic certainly has influenced how he feels, there are also other causes of the depression:

*“It’s not just covid that’s made me… well you could say depressed or sensitive.”*

—Man in his 70s

#### 3.4.2. Need for Increased Person-Centeredness

Participants also said that apart from activities, they would have wanted sessions to focus more on them as persons, and including discussions about their personalities and feelings, and that the intervention could be more tailored to each person.

*“If it’s possible to widen the treatment to more than just activities… but I’m not sure how to put it… It depends on what experiences you have, but more facts or coaching and… pep-talk or what could I say… Perhaps making the treatment more tailored in some way, see what kind of a person you’re working with so to speak.”*

—Man in his 60s

### 3.5. The Power of Being Recognized

Several participants expressed that the calls and the discussions with their therapists were healing and that they looked forward to the calls and missed them when the intervention ended.

#### 3.5.1. Looking Forward to Talking to Someone

Participants said that they looked forward to the calls from their therapist and having someone to talk to:

*“Well, that was good. Because then you had someone to talk to about things and, you awaited that call.”*

—Woman in her 70s

#### 3.5.2. The Healing Power of the Talking Cure

Several participants said that the contact and conversations they had with the therapists were healing and an important part of the treatment:

*“The calls were so very good; the therapist was so trustworthy and sensible… For me it didn’t matter that it was by telephone, for me it was about illuminating, that it enlightened me about my thoughts more than I could do by myself.”*

—Woman in her 60s

Some participants also expressed that they would have liked additional sessions:

*“Perhaps there should be additional calls, a bit more than four weeks. I believe it would have been better with additional calls.”*

—Woman in her 60s

## 4. Discussion

The COVID-19 pandemic has highlighted the need for psychological interventions for depression that can be delivered remotely via telehealth interventions. The present study investigated older adults’ experiences of a telephone-based BA-MI intervention, conducted during the COVID-19 pandemic.

In this study, several participants expressed their feelings about the acceptability of BA as a conceptualization for depression and acceptability for the interventions based on BA, in line with a previous study (Shearsmith et al., 2023). The use of a one-week activity diary with daily registrations was described as increasing the awareness of the link between behavior and mood. The activity planning as well as the follow-up of completed activities were good ways of increasing enjoyable, important, and meaningful activities, in line with BA theory (Dimidjian et al., 2011). However, as reported previously (Shearsmith et al., 2023), the pandemic restrictions made it difficult to engage in many social and cultural activities, increasing the need for problem-solving.

The experiences of MI were divided. Several participants stated that MI was useful in focusing on the activities and gave them a deeper understanding of the potential positive effects of the activities, in line with previous research on MI as a motivational amplifier (Heise et al., 2022; Ji et al., 2021; Renner et al., 2019). Other participants felt that the MI exercises were difficult or uncomfortable, suggesting that individuals vary in their ability to engage in vivid MI. This finding is also in line with previous research showing that although MI interventions are feasible also for older adults (Murphy et al., 2015), the ability to engage in MI differs between individuals, and some individuals seem to lack this ability, known as aphantasia, and results also suggest that the vividness of visual MI may decrease with age (Gulyás et al., 2022). It is also possible that the introductory exercise was too banal, and that the intervention would benefit from using real-life examples from the patients.

Receiving psychological treatment via the telephone was described as similar to face-to-face contact, but not the same. Many participants felt that the telephone contact was less personal and felt that they missed out on non-verbal information. Several participants suggested adding one or more sessions face to face and conducting the rest over the telephone, which could be an alternative when using the intervention in non-pandemic conditions. There are several ways of accommodating this in future trials and in clinical contexts, such as conducting the assessment session and/or the first treatment session face to face with the therapist, either in a clinical setting or as a home visit. Such accommodations might, however, limit accessibility and increase costs compared to telephone sessions only.

It is important to keep in mind that the BA-MI intervention was brief and designed to be delivered as a first-line intervention, and was not designed to replace regular psychotherapy. However, many experienced a need to be seen as whole people with a past and present that they wanted to talk about with their therapist. There is a possibility that the therapeutic relationship was impaired by the manual-based telephone delivery. Furthermore, many participants experienced a need to talk about potential causes that they themselves had identified for their current mood and depression. These discussions were actually raised with the study coordinator conducting the assessment at enrolment, meaning that the participants missed out on discussing this with their therapist. There are two ways to accommodate this when using the intervention in future studies, strengthening the therapeutic alliance and relationship: 1) the assessment could be performed by the therapist, and/or 2) room could be made in the intervention for more conversation about the participants history, them as persons, and their thoughts about potential causes of their depression.

Many participants experienced the therapist contact as healing and important for feeling better, and many said that they looked forward to the calls, in line with previous research (Shearsmith et al., 2023). Some felt that they would have preferred more calls and felt that the intervention and contact ended abruptly. One way of making the ending more gradual could be by adding a booster session, for example, a month after the last session.

This study has several limitations. First, all interviews were conducted over the telephone, which may have led to a loss of non-verbal communication. Second, since the study coordinator conducted the sampling procedure in collaboration with the study therapists after all participants in the treatment group had completed their treatments, the interviews were conducted 1–3 months post-intervention rather than immediately after the intervention, which could limit the participants’ recollections of the intervention. To compensate for this, the interview guide included brief descriptions of the intervention components. Third, the interviews were performed by three different interviewers. Although there was an interview guide, the interviews were semi-structured, which might have made the interviews different depending on who the interviewer was. Additionally, no participant was interviewed by their own therapist or by the study coordinator. This set up was made in order to minimize bias and to maximize the ability for the participants to be honest about their experiences. A fourth limitation is that we did not include any participants who dropped out of the study and declined further participation. We did, however, include participants experiencing no effect of the treatment in the study. A fifth limitation is the predominance of women and participants with Swedish as their native language, which limits the generalizability of the results to older adults in Sweden in general. The sample is, however, representative of the pilot trial, where 80% of the participants were women, and most participants had Swedish as their native language. To increase generalizability, future studies should seek to be more inclusive of individuals with other native languages and cultural backgrounds, as well as including more men.

There are also issues related to how we applied TA that are worth mentioning, since the use of TA differs depending on how the qualitative methodology is viewed (Braun & Clarke, 2020, 2022). A common strategy for the choice of sample size in qualitative studies involves the term “saturation”, where data is collected until no new information is obtained (Braun & Clarke, 2022). We opted for a purposive sampling strategy more aligned with reflexive TA (Braun & Clarke, 2022), choosing participants that we thought could provide variation to maximize our understanding with regard to sex, age, treatment effect, and geography. The purposive sampling was also considered more pragmatic than interviewing all participants in the pilot study. Data were analyzed using thematic analysis based on descriptive phenomenology (Sundler et al., 2019), aligned with a reflexive tradition of TA (Braun & Clarke, 2022). Using this approach, multiple coders strengthen the analysis by offering different or supplementary views, and to maintain reflexivity by questioning statements and pre-understandings of the researchers (Sundler et al., 2019). In a reflexive approach to TA, researcher subjectivity is seen as a tool rather than a problem, and there is no need to reach consensus between collaborating researchers, since coding is subjective and thereby cannot be “accurate” (Braun & Clarke, 2022).

## 5. Conclusions

In summation, the current study indicates telephone-based psychological treatment is not experienced as the same as face-to-face, but is similar in many ways. Manualized delivery via telephone offers many advantages, but also comes with barriers and shortcomings that should be considered by clinicians as well as in future research. Behavioral activation in this intervention was experienced as a good way of increasing activity and improving mood in older adults. Our findings suggest that the therapeutic relationship could be augmented by adding one or more face-to-face sessions, if possible. When using manual-based psychological interventions, one should aim to make them as person-centered as possible, making room for the patients as individuals with both a past and present, rather than just focusing on intervention delivery.

## Figures and Tables

**Table 1:** Interview guide.

Interview Guide
Can you tell me why you signed up for this study? How did you experience receiving the treatment via telephone? A large part of the treatment was about behavioral activation. How did you experience that? You also did exercises involving mental imagery, where you were asked to visualize performing planned activities. How did you experience that? During the treatment, written materials were used during the sessions and between sessions. How did you experience: ○ The patient materials, with information about depressive symptoms and information about the treatment? ○ The activity diary, where you were asked to register what you did and how you felt during one week? ○ The activity planning, where you were asked to plan activities and evaluate the activities? Have you made any changes in your daily life during the treatment? Did you encounter any difficulties during the treatment? Was there anything that made it easier to succeed with the treatment? Did you achieve your goals with the treatment? How has it been since the treatment ended? If you could change the treatment, what would you add or take away? Is there anything we could do to improve the treatment?

**Table 2:** Themes, categories, and summarized findings for each sub-theme.

Themes	Sub-Themes	Summarized Findings
1. Acceptability of BA as concept in relation to depression	Diary increased self-awareness	The use of an activity diary with daily registrations for one week increased the awareness of the link between behavior and mood.
	Activity planning made me more active	The activity planning was described as a good way of getting activities happen and increasing participants’ motivation to perform the activities.
	Follow-up increased motivation	The follow up of completed activities according to the activity plan by the therapist was important for making the activities happen.
	Pandemic restrictions as a barrier for activities	Pandemic restrictions (i.e., social distancing and home confinement) made it difficult to engage in certain activities and was thereby a barrier for activation.
2. MI as motivator or irritator	MI as a useful tool	MI was useful in giving focus towards the goals of the activities and provided a deeper understanding of the potential positive effects of the activities.
	No point using MI	MI can be difficult for some and may feel uncomfortable and pointless.
3. Telephone contact being similar to face-to-face contact, but not the same	Telephone reduced barriers	Remote delivery via telephone reduced barriers for communication related to pandemic restrictions and made remote contact possible for those without access to Internet.
	Disadvantages with telephone contact	Compared with face-to-face interaction, contact over the telephone felt less personal, and non-verbal communication was lost.
	Adding face-to-face contact could improve treatment	The telephone-based intervention could be improved and strengthened by adding one face-to-face session.
4. The importance of being seen as a whole person	The need to discuss causes of depression with a therapist	Many had thoughts about potential causes for their mood and depression and felt a need to talk about these causes with their therapist.
	Need for increased person-centeredness	It is important to make room for discussions about individuals’ history, personalities and needs, and to be flexible about the time needed for these discussions to make the intervention more person-centered.
5. The power of being recognized	Looking forward to talking to someone	Receiving a call from the therapist each week for four weeks felt good and was something to look forward to.
	The healing power of the talking cure	The contact and conversations with the therapists were healing and were an important part of the treatment.

## Data Availability

Data is not sharable due to privacy conditions associated with the ethical approval.

## Abbreviations

The following abbreviations are used in this manuscript:

COVID-19	Coronavirus disease 2019
BA-MI	Behavioral activation with mental imagery
BA	Behavioral activation
MI	Mental imagery
CBT	Cognitive behavior therapy
TA	Thematic analysis
